# What Happens When [Terai] Girls Play? Understanding the Relationship Between Restrictive Gender Norms, Girls' Experiences of Playing Sport in South‐Eastern Nepal, and the Factors That Influence Their Participation

**DOI:** 10.1002/jad.70011

**Published:** 2025-07-02

**Authors:** Sara Begg, Nisha Shah, Mukesh Poudel, Justin Pulford, Sara Parker, Cricket Changemakers (Nepal)

**Affiliations:** ^1^ Liverpool School of Tropical Medicine Liverpool Merseyside UK; ^2^ Sabal Nepal Rajbiraj Madhesh Province Nepal; ^3^ Nepal Cricket Foundation Biratnagar Koshi Province Nepal; ^4^ Liverpool John Moores University Liverpool Merseyside UK

## Abstract

**Introduction:**

Restrictive gender norms globally disproportionately constrain girls, limiting their freedom and mobility while increasing their risk of violence. This study adopts a Youth Participatory Action Research approach to explore how adolescents in Nepal experience these norms in sport, and identify pathways for their positive transformation.

**Methods:**

Twenty‐three adolescent girls from eight government schools in the Terai districts of Morang and Saptari, Nepal, were trained as youth researchers to co‐define the research focus and questions related to gender, sport, and adolescence. They conducted 15 play‐based focus group discussions with 64 adolescent boys and 84 adolescent girls aged 13–19. Outputs were co‐analysed using a framework approach shaped by the youth researchers' lived experiences.

**Results:**

Situating our findings relative to Ecological Systems Theory, we found that girls' participation in sports is limited by restrictions on their free time and mobility, and community and peer “backbiting”. Parents, teachers, and boys in their microsystem reinforced these norms, while schools and sporting institutions provided limited opportunities and uneven resources. However, girls' sporting success emerged as a catalyst for change, generating pride that shifted perspectives on girls' capabilities, rights, and freedoms.

**Conclusions:**

This study highlights the importance of addressing gender norms at a societal level by engaging with the interpersonal interactions that sustain them. It identifies “pride” as a transformative force, supporting evidence that positive norms can drive gender equity. Future interventions should build girls' confidence, engage boys as allies, and increase the visibility of girls' sports to expand freedoms in the Terai.

The situation of women and girls in Nepal reflects the deep‐rooted influence of restrictive gender norms, which shape their opportunities and autonomy, particularly in education, work, and personal decision‐making (Atteraya et al. 2015; Dahal et al. 2022; Lamichhane et al. 2011; Ministry of Health and Population—Nepal, New ERA, & ICF 2023). These norms influence health outcomes, including sexual and reproductive health, mental health, and overall wellbeing (Cislaghi and Heise 2018, 2018, 2020; Cislaghi et al. 2018; Heise et al. 2019). Women and girls face significant barriers to accessing healthcare and health education (Das et al. 2018; Kirk and Sommer 2006; Sen and Iyer 2012; Sheikh and Loney 2018), and their participation in health‐promoting activities such as recreational physical activity is often limited (Furnham et al. 2002; Hayhurst and Del Socorro Cruz Centeno 2019; Sharara et al. 2018). More generally, discrimination, harassment, and restrictions on movement persist across much of Nepal (Dahal et al. 2022; Samuels and Ghimire 2014; Samuels et al. 2017).

While there has been progress, such as rising school attendance, declining acceptance of intimate partner violence, and increased financial decision‐making power for women (Ministry of Health and Population—Nepal, New ERA, & ICF 2023), these improvements are uneven, with regional disparities persisting in secondary school completion rates, unpaid labour burdens, and experiences of gender‐based violence (Dahal et al. 2022; Ministry of Health and Population—Nepal, New ERA, & ICF 2023; Samuels et al. 2017). That there has been progress in some areas does, however, demonstrate that the transformation of harmful and restrictive gender norms is achievable. Some adolescent boys and girls may already be adopting more flexible attitudes towards gender roles, even as older members of their communities continue to uphold traditional norms (Dahal et al. 2022).

Gender norms are formed through a process of gender socialisation, which starts from birth and continues throughout the life course, with adolescence (between the ages of 10 and 19) identified as a key period during which the factors that influence the formation of gender norms have a particularly instructive effect (Lundgren et al. 2019). This socialisation process is deeply embedded in cultural and social practices, however common mechanisms of gender socialisation exist, particularly amongst adolescents.

To understand how these influences shape gender norms, the Ecological Systems Theory first proposed by Bronfenbrenner in 1979 (Bronfenbrenner 1979) provides a useful framework. This Ecological Systems Theory emphasises that adolescent development is shaped by multiple interconnected systems, from immediate interpersonal influences (microsystem) such as family and peers, through the interactions between these settings (mesosystem), such as the relationship between home and school, to external environments that indirectly influence the individual (exosystem), such as local government policies. These all exist within broader societal structures (macrosystem) such as cultural expectations and policy environments. Gender socialisation is therefore not only reinforced within families and peer groups but also maintained through community norms, institutional policies, and broader socio‐cultural structures that shape expectations around masculinity and femininity.

Later developments in Bronfenbrenner's theory further emphasised the dynamic and bidirectional nature of human development, in which adolescents are not only shaped by their environments but also actively influence them in return (Bronfenbrenner 1986, 1994). These systems are interrelated and evolve over time, meaning that gender norms are not fixed but constantly co‐produced through ongoing interactions. This bidirectionality is especially important in adolescence, when individuals begin to assert greater independence and agency. By focusing on this life stage, the current study seeks to explore not only how girls' experiences are shaped by social norms, but also how adolescents may challenge and reshape those norms.

Boys are more likely than girls to uphold gender norms that reinforce inequalities, often embracing masculine roles of toughness and dominance, while girls challenge these norms. Moreau et al. (2019) found boys maintain a “sexual double standard,” valuing male but not female romantic engagement. Studies also show boys receive more behavioural freedom, reinforcing inequalities (Moreau et al. 2019; Yu et al. 2017). Kågesten et al. (2016) highlight that boys, benefiting from gender norms, lack motivation to advocate for equality. Interventions must demonstrate how gender equality benefits them and society (Kågesten et al. 2016). Postpuberty, peer influence increases, particularly for girls, shaping perceptions of acceptable behaviour. Globally, boys are expected to be tough and dominant, while girls are pressured to comply with beauty standards, reinforcing gender inequality (Meyer et al. 2018; Moreau et al. 2019). From an Ecological Systems Theory perspective, this reflects interactions between personal beliefs and peer influence (microsystem), and societal expectations (macrosystem).

Family shape gender norms, with girls facing stricter scrutiny and control over autonomy to conform to societal expectations of purity and obedience (Blum et al. 2017; Moreau et al. 2019). Family dynamics at the interpersonal level, the microsystem, reinforce or challenge norms. Mothers often reinforce traditional roles, while fathers exemplify male authority. For girls, this can include strict control over their autonomy to preserve their “purity” (Stavropoulou 2019). Working mothers in the home can help reduce gender stereotypes, though this does not extend to domestic work, where fathers' involvement often remains low (Ninsiima et al. 2018; Yu et al. 2017).

The mesosystem level of Ecological Systems Theory, the level of institutional and communities in which the adolescent is embedded, explains how schools, sports, and communities reinforce or challenge these norms, and schools play a key role, upholding gender norms through rules, activities, and teacher interactions, often limiting students' perceptions of gender roles (Moreau et al. 2021; Moreau et al. 2021), while school and local government level policies exert influence through the exosystem.

Gender norms are an inherently social construct, and as a result they manifest themselves in all facets of our social lives. Embedded in a community sports programme in South East Nepal, this study specifically uses sports participation as a lens to examine broader issues related to gender norms and adolescence. This study considers the role of proximal processes, the repeated, reciprocal interactions between adolescents and key people in their everyday environments, as central to the formation and transformation of gender norms (Bronfenbrenner and Morris 2007). These processes unfold within real‐world contexts such as homes, schools, and community sports spaces, all of which are sites where gender expectations are learned, contested, or reshaped. By embedding the research within these settings, this study examines how gender norms are experienced and negotiated across microsystems and the mesosystem, and how norms are shaped by the broader exosystem.

Adolescence is widely recognised as a critical period for challenging and transforming restrictive gender expectations (Kågesten et al. 2016). As such, initiatives that engage both boys and girls in gender‐transformative approaches are essential for fostering more equitable norms and supporting sustainable change (Barker et al. 2007; Casey et al. 2018; Kågesten et al. 2016; Lundgren et al. 2013). Sport provides a particularly interesting vehicle for this investigation; sports are spaces where restrictive gender norms can be reinforced, by coaches and teachers (Wilkinson and Penney 2016) and players (Cockburn and Clarke 2002). However, sports are also spaces where restrictive gender norms can be challenged, subverted, and transformed, particularly during early adolescence (Gupta and Santhya 2020; Hayhurst et al. 2015; Santhya et al. 2019).

Darnell et al. (2019) define “sport for development” as: “the processes, theories, and/or ideologies of using sport to attain “positive” social outcomes” (Darnell et al. 2019), such as gender empowerment and economic, health, and peace initiatives. Typically, gender‐norm transformation is a secondary goal in programmes with outcomes related to HIV prevention or youth pregnancy reduction (Grassroots Soccer 2017), or they concentrate on empowering girls (Forde and Frisby 2015) and occasionally fostering gender‐equitable attitudes in both sexes (Hershow et al. 2015; Right to Play n.d.; Woodcock et al. 2012). Mixed‐gender sport activities seldom include gender‐focused content; instead, they often emphasise access for girls; gender equality *in* rather than *through* sport (Chawansky 2011).

However, a context relevant example of sport for development for gender norm transformation is the Parivarthan programme in Mumbai, India. The girls' Kabaddi (a traditional South Asian team sport) and mentoring programme explored the potential of girls' sports participation in transforming gender norms related to mobility and freedoms (Collumbien et al. 2019). The programme focused on empowering girls individually and achieved some success in increasing visibility and mobility of girls in the community, with the role of maternal and peer support key in its success (Cislaghi et al. 2020; STRIVE 2018). The Parivartan boys' programme engaged cricket coaches as role models and influencers of youth behaviour, and adapted the “Coaching Boys Into Men” programme, which aimed to reduce dating violence in the US through role modelling and challenging toxic behaviours led by high‐school coaches (Miller et al. 2016; Miller et al. 2012). The Parivartan programme showed some positive attitudinal changes, and a reduction in negative bystander behaviours.

To develop effective approaches to transforming norms, Cislaghi and Heise (2020) propose action that identifies and strengthens protective norms to achieve positive transformation of social norms, for example norms such as collective community responsibility for each other's wellbeing can be mobilised for joint action on rejecting harmful practices such as Gender Based Violence (Michau et al. 2018; Yancey et al. 2012). Participatory approaches can be an effective tool to this end. Participatory action research (PAR) with young people: “…is an approach to scientific enquiry and social change grounded in principles of equity that engages young people in identifying problems relevant to their own lives, conducting research to understand the problems, and advocating for changes based on research evidence.” (Ozer 2017, p. 89). Using a participatory research approach that involves adolescents and young people as co‐researchers may be a powerful way to better understand restrictive gender norms and identify potential solutions for their positive transformation (Cislaghi and Heise 2018), with youth‐led participatory research increasingly recognised as a way to empower, engage and mobilise youths' expertise to improve health and wellbeing (Ozer 2017). This study applies a novel play‐based Youth Participatory Action Research approach to identify both the nature and impact of restrictive gender norms on sports participation amongst adolescents in South East Nepal, and routes to transformative action.

This study makes several contributions to the field of gender norm research and sport for development. First, it presents the first known account of adolescent girls' lived experiences of sport and its intersections with gender norms in Nepal, offering qualitative insight from a Terai context. Second, it expands conceptual understanding of gender norms in Nepal by evidencing how these norms, widely studied in relation to education, marriage, and health, also profoundly shape girls' ability to engage in sport as a form of social and community participation. In this way, it reframes sport as a social determinant of gendered wellbeing and agency, rather than a peripheral leisure activity. Third, the study identifies “pride” as a potentially transformative social norm that can catalyse changes in gender attitudes and behaviours. Finally, this study advances participatory methodologies by modelling a play‐based Youth Participatory Action Research (YPAR) process that not only engaged adolescent girls in data generation but centred them as co‐analysts, employing tactile, accessible, and context‐sensitive methods that broaden the inclusivity and rigour of YPAR in sport‐based research.

## 1 Methods

### 1.1 Study Overview

**Figure 1:**
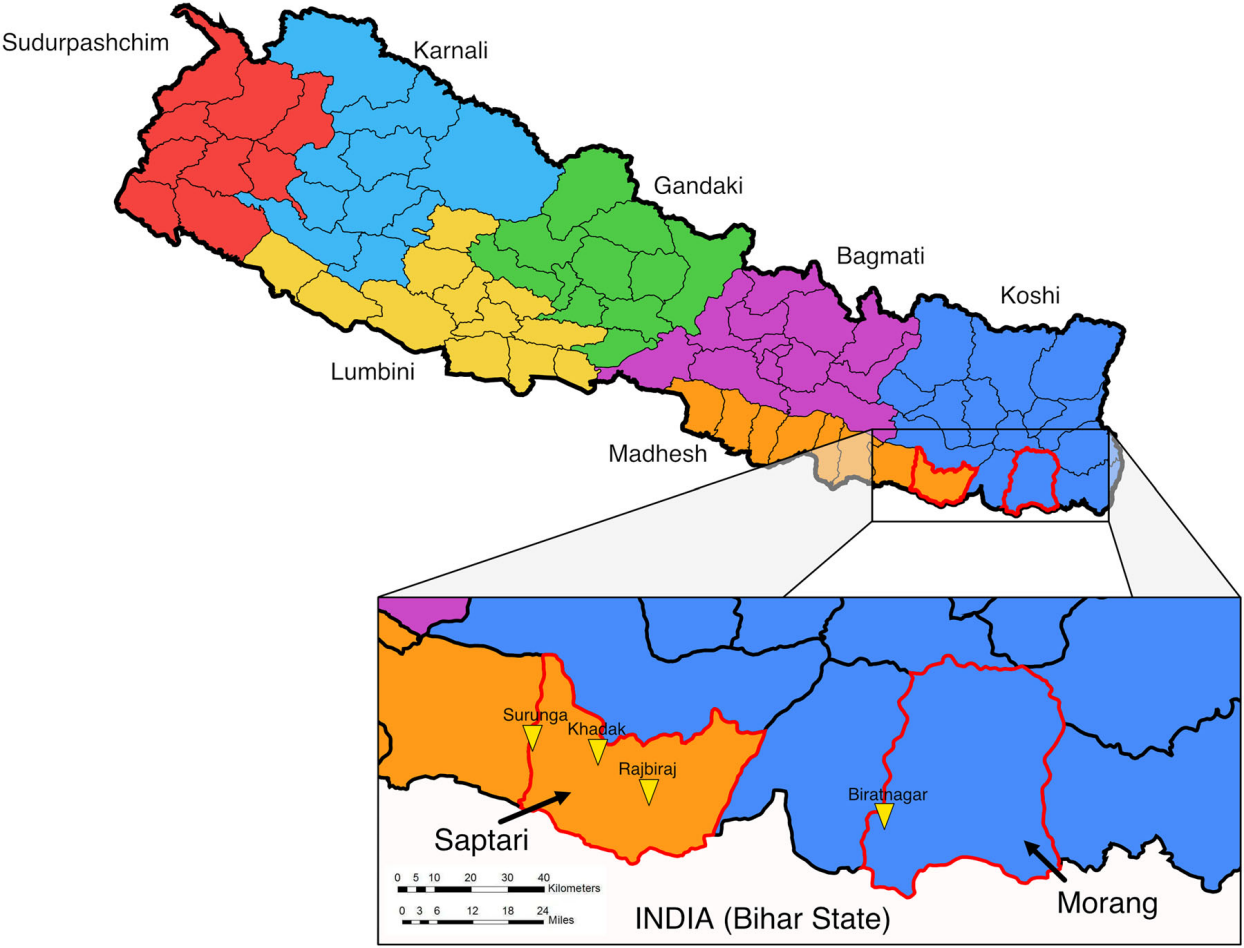
Map highlighting locations of the study districts within Nepal, and the location of cities and municipalities where participating schools were located.

This study is part of the Cricket Changemakers research project. The Cricket Changemakers is a Youth Participatory Action Research project embedded in a community cricket programme in two Terai districts, Morang (Koshi Province) and Saptari (Madhesh Province) (Figure 1), delivered by the Nepal Cricket Foundation, development organisation Sabal Nepal and the Saptari Cricket Association, and supported by the cricket for development organisation Cricket Without Boundaries. In Koshi Province 74% of children complete Lower Secondary education, aligning with the national average (UNICEF 2022). However, 20.5% of women in the province have experienced physical violence since age 15% and 30.9% of girls are married before age 18. Meanwhile, Madhesh Province, entirely in the Terai, reports the highest rates of gender‐based violence in Nepal, with 36.5% of women experiencing physical violence since age 15% and 52.5% of girls married before age 18 (Ministry of Health and Population ‐ Nepal, New ERA, & ICF 2023; Samuels and Ghimire 2021). School completion rates are low, with only 60% of children completing Lower Secondary education, and public harassment of girls remains a widespread issue (Samuels and Ghimire 2021; UNICEF 2022).

In each district, a group of adolescent girls from government schools who currently or would in the future participate in the cricket programme formed a youth research team (“Cricket Changemakers”). Their characteristics are summarised in Table 1. The aim of the wider YPAR project is to identify, describe, and transform a challenge related to gender identified by these Cricket Changemakers. This study represents the first phase of this project and seeks to produce a rich description of the identified challenge to inform the development of activities to create change.

**Table 1:** Participant characteristics.

Variable	Cricket Changemakers		FGD participants	
	Morang (*n* = 9)	Saptari (*n* = 14)	Morang (*n* = 62)	Saptari (*n* = 86)
Age (years)	13.7 (±1.2)	16.2 (±1.7)	12.9 (±1.2)	14.08 (±1.7)
Gender				
Girls	9 (100)	14 (100)	42 (67.7)	42 (48.8)
Boys	0 (0)	0 (0)	20 (32.2)	44 (51.1)
Ethnicity				
Chhetri/Brahmin	3 (33.3)	0 (0)	7 (11.2)	10 (11.6)
Dalit	0 (0)	5 (35.7)	5 (8.1)	10 (11.6)
Janajati	5 (55.6)	6 (42.9)	25 (40.3)	31 (36.0)
Madhesi	1 (11.1)	3 (21.4)	10 (16.1)	25 (29.1)
Muslim	0 (0)	0 (0)	10 (16.1)	4 (4.7)
Other	0 (0)	0 (0)	5 (8.1)	6 (7.0)
School cricket provision				
Yes	9 (100)	4 (28.6)	62 (100)	21 (24.4)
No	0 (0)	10 (71.4)	0 (0)	65 (75.6)

**Figure 2:**
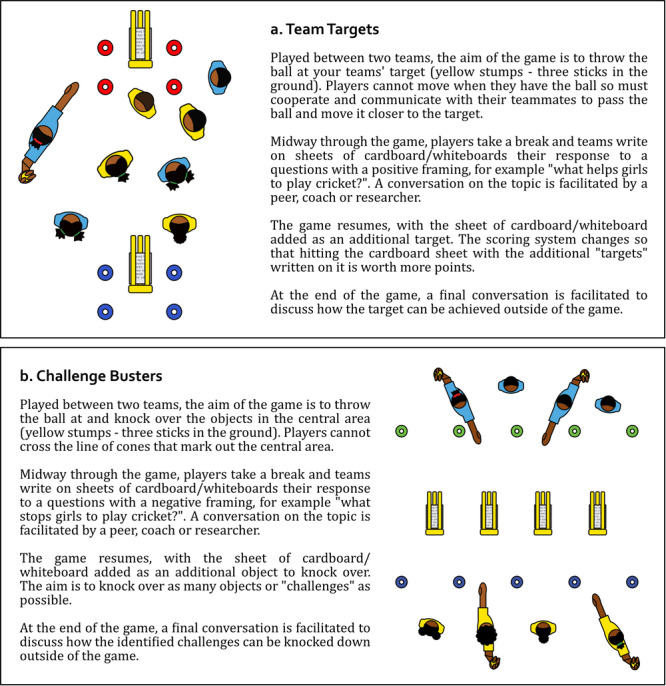
Description of two activities used during the play‐based Focus Group Discussions.

This study took place over 6 weeks in Marg and Falgun 2079 (January and February 2023). At the start of this study period, the Cricket Changemakers took part in a 2‐day workshop to identify a specific challenge related to gender that would form the focus of the research. In this workshop, the Cricket Changemakers in both Saptari and Morang districts played games such as those presented in Figure 2. Through this process they identified restrictive gender norms, in particular those that limit girls' freedoms compared to boys, as the issue of concern (Begg et al. 2024). We then identified research questions which could help us better understand these norms, opting to use girls' involvement in sport as a lens through which we could examine these issues in greater detail. The resulting research questions were:

- What helps girls to play cricket/sport?
- What stops girls from playing cricket/sport?
- What happens when girls play cricket/sport?

We used two complimentary qualitative methods to collect data related to these research questions. An adapted version of PhotoVoice was used to capture Cricket Changemakers' own perspectives and experiences over the study period. To gather input from school‐age peers we also conducted a series of play‐based Focus Group Discussions with adolescent boys and girls.

Sixty‐four adolescent boys and eighty‐four adolescent girls took part in fifteen play‐based Focus Group Discussions over six events (Table 1). Of those invited to take part, twenty‐eight declined to participate or did not attend, and four attended the session but were not able to participate as their parents had not completed the consent documentation.

### 1.2 Data Collection Methods

#### 1.2.1 Photo Capture for Modified Photo Voice

Cricket Changemakers were issued with or used their own smartphones to capture images and videos they felt answered or provided insight into their identified research questions, based on their own experiences during the 1‐month data collection period, in a modification of PhotoVoice (Amos et al. 2012). The purpose of this method was to document experiences for later recall and exploration (Gioacchino and Williams 2016), and Cricket Changemakers were encouraged to capture content whenever they felt inspired to do so. Photos and videos were gathered using a dedicated KoboToolbox form, which asked Cricket Changemakers to capture the photograph or video, confirm all people who appeared had provided verbal consent, and document the location the photograph or video was taken (Kobo 2023).

#### 1.2.2 Play‐Based Focus Group Discussions

Play‐based Focus‐Group Discussions were conducted in single gender groups of 8–12 young people from the same school. Participating schools were the same 8 schools (4 per district) as those attended by the Cricket Changemakers. Participants in the Focus Group Discussions were recruited 1 week before the data collection events. Cricket Changemakers and sport focal‐persons at each school were briefed to invite 12 boys and 12 girls to each event, including cricketers and non‐cricketers, from classes 7, 8, and 9 (ages 13–19). Parents and guardians were engaged, and completed written informed consent, gathered either in advance of or on the day of the event. Participants provided verbal assent to take part when they arrived and registered their attendance at the event and were reminded of the right to withdraw.

**Figure 3:**
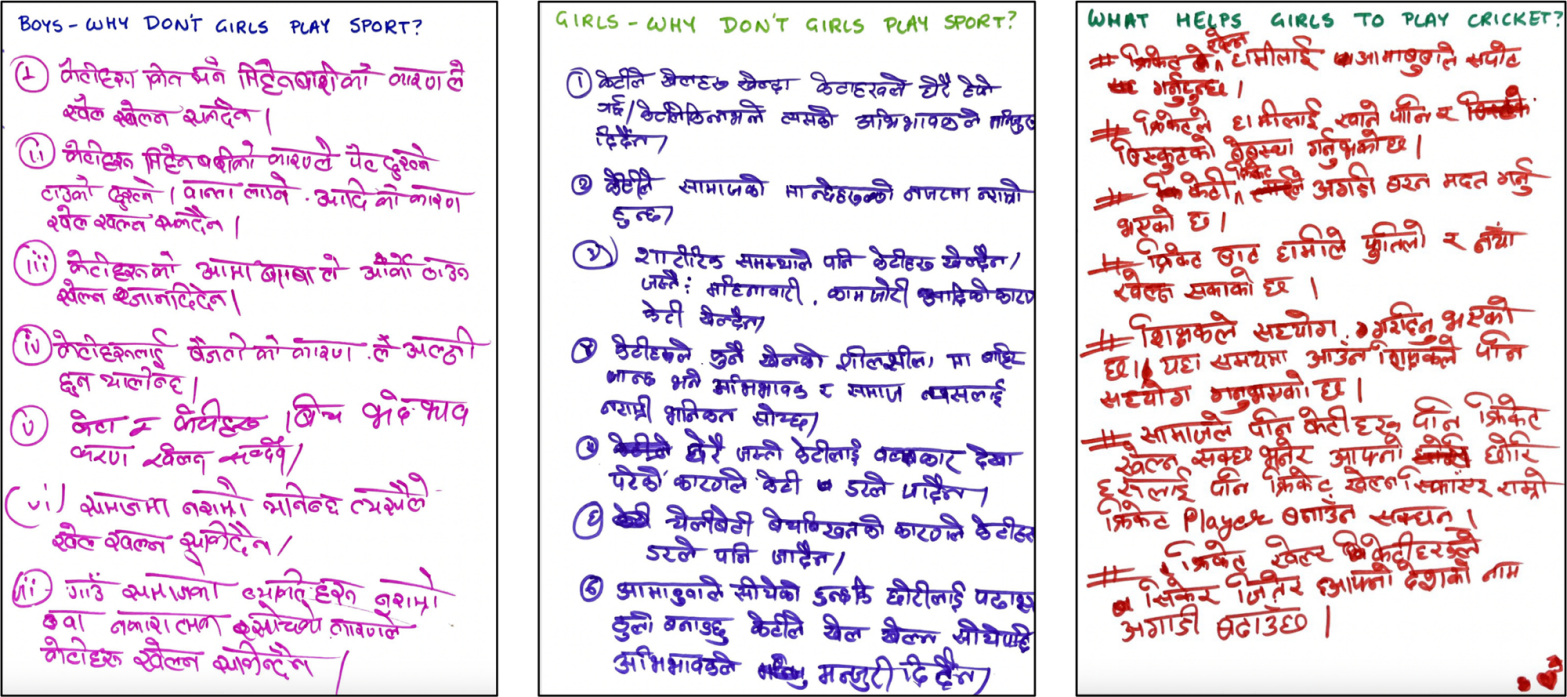
Example raw data collected before division into individual statements.

Conventional FGDs approaches present significant limitations when applied to youth, particularly in sports‐for‐development contexts. They are often constrained by logistical challenges, with sports coaches reluctant to allocate time and space for research activities, and participants showing limited interest in tasks requiring literacy or prolonged concentration (Luguetti et al. 2022; Spaaij et al. 2018). We have found, through analysis of the methods used in this study, that a play‐based approach contributes to collaboration, networking, and communication among participants, as well as supporting the development of collective power (Begg et al. 2024). We therefore used an integrated approach with cricket‐related games providing stimulus for discussion points in the FGD (Figure 2). In each activity, outputs from the discussions were captured on sheets of A4 cardboard. A total of seventy‐four A4 cards worth of data, with 310 statements, were collected and used in the analysis (examples, Figure 3). These Focus‐Group Discussions were conducted in a range of outdoor environments, including school playgrounds, public fields, and cricket grounds, as part of a 2‐h long event. 30 to 40 min of each event was spent in active discussion.

The events were overseen and coordinated by a UK‐based PhD researcher who is also an accredited cricket coach. In both locations she was supported by a Nepali research assistant and a Nepali cricket coach. Between 4 and 8 Cricket Changemakers attended each event, where they supported the explanation of games, kept score and managed games, and facilitated the discussion opportunities. Cricket Changemakers undertook a 1‐day workshop before facilitating these events to practice how the games run, how to encourage their peers to discuss the identified topics together, and capture their ideas on the provided materials.

### 1.3 Data Analysis

**Figure 4:**
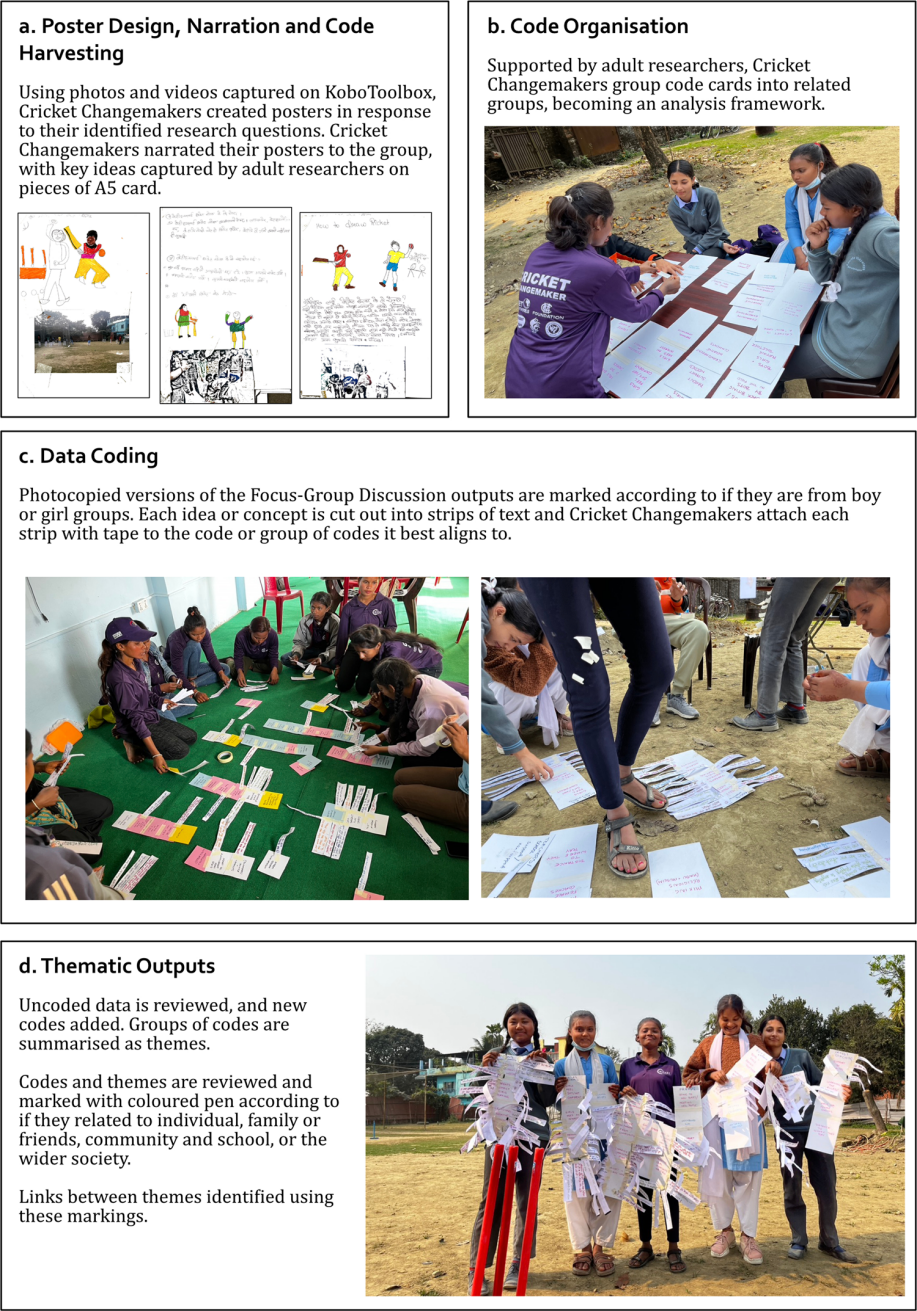
Interactive, multi‐stage approach for framework analysis.

Analysis was conducted through an inductive framework approach (Goldsmith 2021). Site‐specific analysis was conducted, with the Cricket Changemakers in Saptari and Morang districts participating in 2‐day workshops to analyse the data that they had collected. Analysis was conducted in multiple phases which were designed to emphasise the voices of the Cricket Changemakers in the development of the framework, codes, themes, and descriptions of themes and the relationships between themes, and was informed by the approach applied by Liebenberg et al. (2020). This highly interactive and tactile approach is visually summarised in Figure 4 (Jackson 2008).

An initial framework was generated through the analysis of videos and photos captured by the Cricket Changemakers on KoboToolbox. Cricket Changemakers used photos and videos they felt were most significant to design a poster to summarise their own response to the research questions (Figure 4a). These posters were presented to the group, and prompts used in Photo Voice analysis were used to encourage deeper exploration of the significance of the poster content (Amos et al. 2012). As the discussion progressed, facilitators implemented “code harvesting”, writing down potential codes live on A5 cards. The Cricket Changemakers worked together to organise these cards into groups of related codes, to form a framework for the next phase of analysis (Figure 4b).

The resulting framework was used to analyse the outputs from the play‐based Focus Group Discussions. Each line or statement captured on the outputs was marked based on if it came from a boy or girl group, and cut out as an individual “strip” of data (examples attached to cards in Figure 4c, d). Cricket Changemakers worked together to attach each strip of data to the part of the framework they felt it most strongly matched with (Figure 4c). Any data left uncoded at the end of this process was collected. Cricket Changemakers discussed if this data was significant, and, if so, how the framework should be modified to include it. A final phase of analysis used coloured pens to mark connections between themes, based on who or where the themes were connected to: the individual, family or friends, community and school, or the wider society (Figure 4d).

### 1.4 Data Presentation

**Figure 5:**
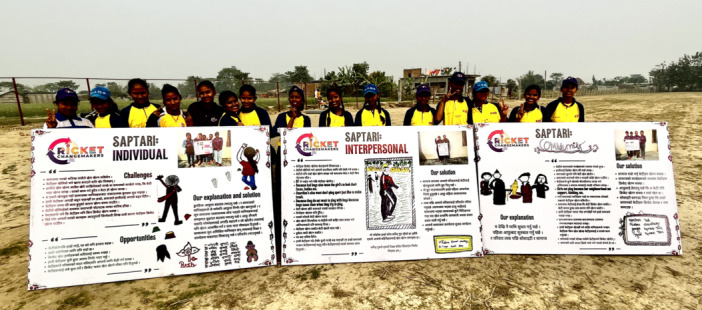
Research posters as shared at the Equality Cup competition on 8th March 2023.

To produce narrative summaries of the key findings, the Cricket Changemakers selected key quotes and images from the analysis process and designed and developed posters aligned to the individual, interpersonal, community or societal level, with the identified themes that connected to these levels of actions summarised or illustrated. The purpose of collecting findings and themes into these groups was to inform multilevel action in the planning phase which was to follow, which has been identified as key in social norms transformation interventions (Cislaghi et al. 2018). Examples of posters from Saptari as shared at a public engagement event are presented in Figure 5.

### 1.5 Data Integration

Data from the two locations was integrated by the PhD student using a framework which mapped illustrative data on a grid with themes from the co‐analysis process on one axis and where the data relates to influencing factors or girls' experiences on the other axis (illustrative quotes data mapping in Supporting Information A). Data, which had been co‐analysed in the original Nepali, was translated by a Nepali research assistant into English to allow this data integration.

### 1.6 Ethical Considerations

YPAR presents distinct ethical considerations, particularly around power dynamics, informed consent, and the well‐being of adolescent participants (Löfman et al. 2004; Ozer 2017). In this study, adolescent girls took on active researcher roles, making it essential to ensure co‐ownership of the research process while safeguarding their privacy, agency, and emotional safety. Ethical challenges included the risk of coercion in peer‐facilitated discussions and the sensitivity of addressing restrictive gender norms within settings marked by social hierarchies and unequal power relations. To mitigate these risks, we implemented robust informed consent procedures, ongoing reflexive dialogues around positionality, and participatory approaches to data analysis, supporting both ethical integrity and youth agency (Liebenberg et al. 2020; Löfman et al. 2004). Given the public visibility of girls' participation in sports‐based research activities, additional care was required to consider the social norms that could increase their vulnerability. In response, we collaborated closely with partner organisations and youth researchers to identify spaces that were both accessible and acceptable to participants, reflecting the context‐sensitive and inclusive spirit advocated in YPAR literature (Ozer 2017; Teixeira et al. 2021). Applying these playful, sports‐based research activities was, however, vital to address concerns around extractive and discordant research methods applied in sport for development (Luguetti et al. 2022).

## 2 Results

### 2.1 Findings Overview

Gender norms significantly shape the environment for girls' participation in sports, influencing behaviours and attitudes at multiple levels of Ecological Systems Theory (Bronfenbrenner 1979). The themes generated by the girls who co‐analysed the data highlight how restrictive gender norms function across different system levels: at the microsystem level, shaping individual beliefs and direct interactions with family and peers; at the mesosystem and exosystem levels, influencing community norms, school environments, and institutional policies; and at the macrosystem level, where broader societal expectations and policies reinforce or challenge these norms.

We found that both adolescent boys and girls in Morang and Saptari believe that gender norms in Nepali society are the principal factor that hinder girls' participation in sports, limiting their free time and mobility while exposing them to violence and the risk of violence. These norms are enforced by key groups of individuals through their interpersonal interactions. Parents were the primary enforcers of restrictive norms, while adolescent boys exhibited harmful behaviours stemming from these norms in the form of bullying and teasing. However, both girls and boys expressed a desire to be agents of change. Furthermore, we found that the pride girls, their families, and the wider community felt from their sporting success could transform individuals, interpersonal relationships, the community, and society's perspectives on girls' capabilities and their rights to social freedoms.

### 2.2 Advocates for Change

Gender norms played a significant role in creating a challenging environment for girls' participation in sports, influencing behaviours and attitudes. However, there was a presence of support and advocacy for transforming girls' opportunities and involvement in sports from both adolescent boys and girls, particularly in Morang province. For examples, boys captured perspectives about the role of family and the community in upholding girls' rights to play “Family should not engage girls in household activities so that girls get time to play cricket.” and “We should raise awareness [in] our community that girls are also human and should provide equal rights”. These beliefs and attitudes challenge the prevailing norms, and suggest the existence of unresolved tensions or untapped potential for change.

It is within this context that we describe the behaviours, beliefs and attitudes associated with the established norms and examine the resulting experiences of adolescent girls, recognising that social norms do not necessarily reflect individuals' beliefs or attitudes, but that they often act as a strict enforcer of behaviours that reflect the social norm.

### 2.3 Gender Norms and Girls' Roles

#### 2.3.1 Girls as Homemakers (Microsystem and Mesosystem Influences)

Norms regarding gender roles positioned girls as responsible for household chores, restricting their mobility and leisure time. Families reinforced this expectation, as expressed by boys: “If girls play, when will they learn to cook food?”. The role of the microsystem — family and peers — in shaping these norms is evident, with boys using societal expectations to exclude girls from sports: “People tease the girls, ‘Do not play sports, you should focus on household work.’” Beyond the immediate family and peer group, mesosystem‐level pressures — such as widespread community norms — further reinforced these expectations. Even when girls' families were supportive of their participation in community sports, external social pressures discouraged their involvement.

As a result, these gendered norms restricted girls' movement and mobility. Parents were hesitant to allow their daughters to travel alone to sports venues due to concerns about societal scrutiny and potential negative comments from the wider community. The combination of microsystem influences (family and peer attitudes) and mesosystem pressures (broader community expectations) contributed to these restrictions, reinforcing the belief that girls should remain at home rather than participate in sports.

#### 2.3.2 A Secure and Pure Future (Microsystem, Mesosystem, and Macrosystem Influences)

Girls' participation in sports was constrained by concerns over reputation, marriageability, and future security, shaped by influences at multiple levels of the Ecological System. Within the microsystem, parents dismissed sports as part of a future for girls: “Parents believe that boys can achieve fame [through sports], but girls cannot”. Some girls were encouraged by their parents to prioritise schoolwork and studies to secure a good job, while others were discouraged from focusing on academics and instead emphasised household duties and marriage preparation: “We girls also want to study and become successful individuals, but our community believes that girls don't need to study and should focus on household chores.”. These microsystem‐level restrictions reflect how parents enforce macrosystem‐level societal expectations regarding gender roles and future security.

The result of these norms was restrictions on girls playing due to the perceived risk that it would hinder their chances of getting married, making them less desirable and compromising their purity, for example if they are seen wearing “unsuitable” clothing or participating in mixed sport, as identified by boy students from Morang: “Boys and girls are playing [together], and other people interpret that negatively.”. In addition, getting married was seen as a reason for girls who played sports to discontinue their participation, as they came under the influence of their husband's family: “[Girls stop playing] due to the notion that girls move to their husband's house after marriage.”. This highlights how microsystem influences (family expectations), mesosystem pressures (community beliefs), and macrosystem norms (broader cultural expectations around purity and marriageability) collectively shape the restrictions on girls' participation in sports.

#### 2.3.3 Girls' (In)Capability (Microsystem, Mesosystem, and Exosystem Influences)

Perceptions of girls as weak or prone to injury further reinforced restrictive gender roles. While there was support and advocacy for girls' right and opportunity to engage in sports, there existed a divergence between girls' and boys' perspectives regarding girls' abilities. Girls strongly affirmed their capacity to play sports, while boys more commonly described girls as “weak in nature” and prone to injuries. These concerns reinforced broader macrosystem norms that tied girls' future roles to homemaking and marriage, as reflected in parental anxieties: reported by participants “Who will marry you if you are injured or disabled while playing?”.

At the microsystem level, these attitudes were reinforced within families and peer groups, where expectations around girls' physical abilities discouraged participation. At the mesosystem level, institutional influences such as teachers and community figures further shaped these norms. For example, a student group in Saptari explained: “In any competition at school, if we show interest in participating, teachers do not allow girls to participate.”. Conversely in Morang, where community sports programmes were more developed, some sports focal teachers played a role in shifting these perceptions, highlighting the potential for exosystem interventions (institutional policies and programming) to challenge restrictive gender norms.

In both Saptari and Morang, when girls challenged these beliefs and attitudes about their capabilities by developing and showcasing their cricket skills, it had a profound impact on others' perspectives, including teachers and community members. This shift underscores the importance of institutional support (exosystem) and positive role models (microsystem and mesosystem) in changing perceptions of girls' capabilities in sport.

#### 2.3.4 Normalisation of Violence (Microsystem, Mesosystem, and Exosystem Influences)

Harassment and violence against girls in sports were widespread, highlighting peer‐enforced norms within the microsystem: “When girls play cricket, boys jump over their bodies. Boys beat girls… They push the girls around on the playground, and it makes the girls feel ashamed.”. In addition to experiencing harassment and violence from their boy peers, girls emphasised the role of their “boyfriends” in controlling their activities, including prohibiting them from participating in sports. These restrictions illustrate microsystem‐level gendered power dynamics, where interpersonal relationships further limit girls' autonomy.

At the mesosystem level, the persistence of these behaviours reflected community acceptance of gendered violence. Safety concerns further restricted girls' participation, particularly in Saptari: “Many girls are raped, so due to the fear for their safety, girls avoid participating in sports.” and “Due to girls trafficking, girls fear going out.” This fear reflects exosystem influences, where societal structures fail to protect girls from harassment and mobility restrictions are imposed as a protective measure, and meant that even parents who permitted girls to play required daughters to be home before dark.

#### 2.3.5 Distribution of Resources (Mesosystem and Exosystem Influences)

The availability of playing spaces, coaching, and equipment in the context of limited resources, particularly for cricket constrained both boys' and girls' participation in Morang and Saptari, especially within government schools. The limited resources that were accessible were unevenly distributed, with boys receiving priority access. The Cricket Changemakers from Saptari highlighted this issue in their analysis, producing results posters that depicted the unequal distribution of coaching resources. One caption read: “In this picture, our coach Dharmendra is coaching girls to play cricket. He is alone in coaching girls.”. This was mirrored in broader discriminatory behaviours described by participants as “discrimination between boys and girls.”, with examples raised including differential treatment of parents paying for boys to attend private schools while girls were enroled in government schools.

These disparities reflect institutional‐level barriers at the exosystem level, where systemic inequalities and structural discrimination restrict girls' access to sports. Schools played a significant role in reinforcing these disparities, either through direct exclusion or a lack of adequate support for girls' participation in sports.

### 2.4 Pride and Transformation

An exception to the restrictive gender norms that hindered girls' participation was the societal emphasis on national pride and the desire to bring honour to Nepal, the community, and the family through sporting achievements. This sentiment was particularly strong in Morang, where girls in the community had gone on to represent the district, province, and Nepal in cricket. Drawing on personal experiences, they explained how girls' success contributed to changing perspectives and altering their social standing, with girls who play explaining: “If we play cricket, we gain respect. When I was a district player, many people recognised us.”.

The transformative power of pride was closely tied to the visibility of girls' accomplishments and demonstrated capability. Seeing girls succeed influenced various stakeholders within the microsystem (families, peers, teachers) and mesosystem (schools, local communities), making them more supportive of girls' participation in sports. This was evident in families' shifting attitudes, as girl students from Morang highlighted: “When we play well and receive awards from the school, our parents feel proud of us”. Achievements in sports had a positive influence on boy peers as well, as they responded more favourably and refrained from bullying. Boys in Saptari stated, “If girls play well, boys perceive it positively and refrain from engaging in bullying.”. The impact extended to girl peers, who were motivated to play cricket watching other girls' participate: “After seeing girls playing, it inspires other girls to engage in cricket.”. This shift highlights microsystem‐level change (individual success fostering interpersonal support) and mesosystem influence (broader community acceptance of girls' participation in sports). It also suggests that institutional (school) recognition can help challenge restrictive norms and lead to exosystem‐level policy shifts that promote gender equity in sports.

## 3 Discussion

This study highlights how gender norms in Nepal shape Nepali adolescent girls' lives, particularly impacting their mobility, exposure to violence, discrimination, and access to opportunities like sports. Applying the Ecological Systems Theory (Bronfenbrenner 1979, 1986, 1994), we see that these norms operate across nested systems, limiting girls' freedom, emphasising domestic roles, and prioritising their future as caregivers and spouses, restricting social and community engagement. This approach highlights how adolescent development is shaped through dynamic interactions between individuals and their environments, with gender norms reinforced or contested across microsystem (e.g., family and peers), mesosystem (e.g., school‐community relationships), exosystem (e.g., institutional policies), and macrosystem (e.g., cultural beliefs) levels (Bronfenbrenner 1994; Bronfenbrenner and Morris 2007) (Bronfenbrenner 1994; Bronfenbrenner & Morris, 2006). These norms restrict their social and community engagement, foster environments where gender‐based violence is normalised, and contribute to boys receiving preferential access to resources and opportunities.

### 3.1 Barriers to Girls' Sports Participation Across Ecological Systems

Mobility restrictions in the micro and mesosystem (interpersonal and community levels) prevent girls from freely accessing sports, reflecting findings from both Nepal and nearby states in India. This includes general mobility restrictions, restrictions on movement in the evenings, and less frequently direct restriction on participation in activities such as sports (Ade et al. 2022). Our research suggests that while girls may not be directly banned from participation in sport, restrictions placed on their time and movement essentially excludes them from these kinds of social community activities. The normalisation of harassment in public spaces — reported in our study and in Uttar Pradesh (Ade et al. 2022) — can further increase girls' isolation and limit their social networks as they withdraw or are kept out of public spaces, which can lead to significant challenges for health and wellbeing including increased risk of depression and anxiety.

Gendered harassment and violence in the micro and mesosystem shape girls' everyday experiences, with threats from peers and even romantic partners controlling their activities. This aligns with previous studies in Nepal, which report high levels of gender‐based violence from families, peers, and intimate partners (Amin et al. 2014; Atteraya et al. 2018; Gyawali 2020; Plan International 2015). This harassment contributes to negative mental health outcomes, including increased rates of sadness, lack of confidence, and depression (Samuels et al. 2017), with this gendered distress linked to Nepal's high rates of female suicide, with suicide accounting for 63% of deaths among women aged 15‐29 (Kasaju et al. 2021).

Gendered disparities persist at the institutional (exosystem) level, mirroring trends in wider Nepali society where girls are sent to government schools while boys attend private institutions (Hatlebakk 2017; Khanal 2018; Samuels et al. 2017). Our study highlighted access to and provision of sporting resources, facilities, and instruction as another example of inequality, reinforcing global patterns of gender discrimination in sports (Fowlie et al. 2021), including across South Asia (Laar et al. 2019; Nanayakkara 2012; Rao 2010), though recent investment in women's cricket signals a potential shift in attitudes, with increasing recognition of sports as a space for contesting gender norms (Thomson et al. 2023). The exosystem enforces these disparities through school and institutional policies that deprioritise girls' sports participation.

### 3.2 Gender Norms and Emerging Opportunities for Change

Our findings indicated a discrepancy between the behaviours dictated by gender norms and the personal attitudes and beliefs of many adolescent participants, suggesting a moment of transition. As social norms impacting adolescent girls in Nepal continue to evolve, such as decreased acceptance of child marriage (Samuels et al. 2017) and increased emphasis on girls' education (Samuels and Ghimire 2014), there is an opportunity for continued progress. The Parivarthan programme explored the potential of girls' sports participation in transforming gender norms related to mobility and freedoms (Collumbien et al. 2019). While the programme achieved some success, it was limited to a single gender approach (Bankar et al. 2018). Similarly, in Bihar and Uttar Pradesh, India, participation in youth group activities improved girls' gender norm related attitudes, however it had little effect on boys, or even reinforced existing biases (Patel et al. 2021). Our findings align partly with this: girls involved in community sport became more aware of their rights, while in areas with active girls' cricket programmes, boys were more supportive of girls' sports. Studies with very young adolescents in Nepal indicate that gender‐transformative approaches engaging both boys and girls lead to greater change, particularly when paired with family and community interventions (Lundgren et al. 2013). Given the greater frequency of supportive attitudes displayed by boys in areas where girls were actively involved in cricket, our research suggests that well‐resourced, mixed‐gender or parallel sports programmes fostering interaction can be a route to positively influencing adolescents' gender attitudes including by challenging interpersonal and community‐level gender norms in the microsystem and mesosystem. These findings align with Bronfenbrenner's assertion that adolescents are not only influenced by their social environments but also capable of shaping them; a bidirectional process central to the ecological model (Bronfenbrenner 1986).

### 3.3 The Role of National Pride in Transforming Norms

Transforming individual attitudes is not sufficient to transform social norms (Cislaghi and Heise 2020). Our study identifies national pride as a protective norm that can be mobilised to drive change. Success in sports allowed girls to challenge negative perceptions, with communities position on girls' participation changing as girls gained recognition: “If we play cricket, we gain respect.” The exosystem and macrosystem influence here is significant — when sports success is linked to national identity, it can catalyse broader societal shifts. This reflects the higher‐level influence of the macrosystem as conceptualised in Ecological Systems Theory, where cultural values, media narratives, and national identity can shape and be shaped bylocal social norms and institutional practices (Bronfenbrenner 1994).

National pride has been conceptualised as having two origins: pride generated by individuals based on national successes, and pride instilled by the state and its institutions. The latter, described as a social norm (Fabrykant and Magun 2016), offers a strategic opportunity for gender norm transformation. If national pride is an underlying driver of support for girls' sports in Nepal, it could be leveraged as a transformative mechanism at the macrosystem level. However, the effectiveness of this strategy may diminish over time as education levels rise and national pride shifts towards more grounded, individual evaluations of national progress (Fabrykant and Magun 2016). Understanding the role of a more localised pride may be necessary to fully realise this potential lever for change.

### 3.4 Limitations

While we sought cricketers and non‐cricketers to take part in each Focus Group Discussion, our participant sample may have been biased towards those who were already supportive of or felt positively towards sport, particularly given the sports‐based nature of the activities. Conversely, this may have helped participants connect more easily with their observations and experiences of girls' participation in sport.

There may also have been a bias in whose voices were documented during discussions, as responses were written collectively on A4 cards. Where Cricket Changemakers led the documentation, we are more confident that a full range of views was captured. However, where participants wrote their own responses, data may have skewed towards the most confident or literate group members.

The composition of the research team may have further shaped the narrative produced. The PhD student overseeing the research was known to many participants and supported cricket coaching in both locations. While this familiarity may have fostered trust, it may also have introduced confirmation bias, with participants potentially expressing support for girls' sport even if it was not a personally held belief. In addition, the Cricket Changemakers were largely from ethnic groups more permissive of girls' involvement in sport, which may have influenced facilitation, analysis, and interpretation in ways that differ from the experiences of participants from other groups.

## 4 Conclusion

This study offers original conceptual, empirical, and methodological contributions to the study of gender norm transformation in adolescence. Conceptually, it reframes sport as a site and lens through which to understand and intervene in gendered socialisation processes, highlighting its role as a determinant of girls' social inclusion, safety, and agency. Empirically, it provides the first documented exploration of adolescent girls' experiences in sport within the Nepal context, revealing how sport‐related pride can serve as a protective norm capable of disrupting harmful gender expectations. Methodologically, the study models an innovative YPAR approach that was not only youth‐led in data collection but deeply participatory in its co‐analysis phase. The use of play‐based and tactile co‐analysis processes allowed adolescent girls to meaningfully contribute to framework development, making the research process more inclusive, contextually grounded, and aligned with the lived realities of the participants.

Baum (2006) note that PAR can identify and strengthen positive norms within a community's value system, while previous research on social norm transformation has identified that social norm interventions often ignore protective norms and attitudes (Cislaghi and Heise 2018). By adopting a playful participatory approach, we gathered direct insights from adolescent boys and girls on their lived experiences and observations. Driven by adolescent girls and young women from the communities, we used sport as a lens to understand how gender norms shape girls' lives and to identify actionable pathways for change. These descriptions add to existing research on how gender norms impact girls' broader life experiences in Nepal.

To challenge deeply rooted norms, interventions must work across multiple levels, addressing societal‐level beliefs while engaging in interpersonal interactions that uphold these norms. We have identified national pride as a positive and protective norm that can be leveraged across these levels to transform harmful gender norms. Alongside this, preliminary evidence suggests that mixed‐gender sports participation may provide a relational approach to gender norm transformation.

To further explore these opportunities, we propose a series of actions to be implemented and evaluated in the next phase of research, with the aim of informing policy and practice. First, we will focus on building girls' confidence and foundational skills in sport, particularly cricket, through peer‐led coaching and both inter‐ and intra‐school competitions. Second, we plan to engage boys and girls in joint cricket activities where open discussions on gendered restrictions can take place, alongside efforts to directly address and challenge behaviours related to gender‐based violence, including bullying and harassment. Finally, we will work to enhance the visibility of girls' sport within the community by organising local competitions, matches, and tournaments, and by producing high‐quality media content that celebrates and promotes girls' participation.

## Conflicts of Interest

The authors declare no conflicts of interest.

## Supporting information

What happens when girls play_SMA.

## Data Availability

The data that support the findings of this study are available from the corresponding author, SB, upon reasonable request.
