# Supplementary material for: What Happens When [Terai] Girls Play? Understanding the Relationship Between Restrictive Gender Norms, Girls' Experiences of Playing Sport in South‐Eastern Nepal, and the Factors That Influence Their Participation
Source: J Adolesc. 2025 Jul 2;97(7):1895–909. doi: 10.1002/jad.70011 (PMC12493011; doi:10.1002/jad.70011)
Supplement: Supplementary file 1 — What happens when girls play_SMA. [file JAD-97-1895-s001.docx]

Supplementary Material A: Illustrative Quotes in Unified Matrix

|  |  | Behaviours, beliefs, and attitudes | | | | | | Experiences | | | | |
| --- | --- | --- | --- | --- | --- | --- | --- | --- | --- | --- | --- | --- |
|  |  | Individual | | Interpersonal | | Community | | Mobility and Freedom | Violence and Discrimination | | Opportunity and Access | |
| (Restrictive) Gender Norms | Girls as homemakers | Due to household work and studies girls cannot concentrate on cricket. | | If girls focus on playing they cannot do their household activities and their parents also do not support them for playing | | Due to negative comments by the community their parents do not permit girls to play | | Due to household chores, girls do not have the same capacity as boys | Because people tease the girls “do not play sport, you do household work” | | Girls do not play cricket because they do not get support and opportunities… due to the community belief that girls are only supposed for household activities | |
|  | A secure and pure future | Due to the fear of physical illness they do not play.  Thinking of girls that they lose their beauty  Due to the fear of bad impression, they do not play | | Parents think that success is only through studies so parents do not allow girls in sports  Thinking of parents that if girls go out for playing their future will not be secured | | If any girls gets jobs the community takes it negatively also thinking that girls wearing short clothes is a negative thing | | Girls go to their husband's house after marriage so they do not play | Due to their chest movement and others looking in a negative way, they do not play.  Girls should not be involved with bad company  Girls do not play due to early marriage | |  | |
|  | Girls’ (in)capability | If we are playing cricket we are physically and mentally strong  Girls are not as strong as boys (F)  Girls are weak in nature (F) | | If any girl play well other girls get motivated  Due to their menstruation they cannot play (M)  Girls are not as strong as boys (M)  Girls do not play sport because girls is very weak (M) | | If girls play well, it can change the community's perception and the concept of boys. | |  | Boys tease during girls playing cricket | |  | |
|  | Normalisation of violence | Due to fear from boys girl do not play cricket | | Boys tease and bully girls while playing sport  Girls do not play due to restriction from boyfriend | |  | | Most of the girls are raped so due to the fear of their security they are not involved in sports | When girls play cricket then boys jump over girls body. Boys beat girls. Boys bullying the girls. | |  | |
|  | Distribution of resources | Due to lack of support and opportunities we do not play cricket | | Due to discrimination between boys and girls by family  Girls do not play due to high opportunities for boys from the family | | There are only cricket shoes for boys in Nepal | |  |  | | Because there is not any playground in our society where girls can play  Teachers only support boys playing cricket | |
| Other Norms | Pride and transformation | If girls can earn name and fame in the community through sports other girls also get motivated | Girls can support their family through sports  If girls play well and the school awards us then our parents feel proud of us | | If girls can earn name and fame in the community through sports other girls also get motivated  If we play cricket we should give respect just like I was a district player then many people can know us | | If girls perform well at the international level, then she keeps pride of the nation |  | |  | |  |
